# Acute GLP‐1 infusion in male adults lowers circulating angiotensin II without changing angiotensinogen, ACE, ACE2, or angiotensin‐(1–7) concentrations

**DOI:** 10.14814/phy2.70940

**Published:** 2026-06-05

**Authors:** Ali Asmar, Muskaan Akram, Charlotte M. Sorensen, Jesper K. Andresen, Boye L. Jensen

**Affiliations:** ^1^ Department of Clinical Physiology and Nuclear Medicine, Bispebjerg and Frederiksberg Hospital University Hospital of Copenhagen Copenhagen Denmark; ^2^ Department of Clinical Physiology and Nuclear Medicine Rigshospitalet, Copenhagen University Hospital Copenhagen Denmark; ^3^ Department of Clinical Medicine, Faculty of Health and Medical Sciences University of Copenhagen Copenhagen Denmark; ^4^ Department of Biomedical Sciences University of Copenhagen Copenhagen Denmark; ^5^ Department of Cardiovascular and Renal Research, Institute of Molecular Medicine University of Southern Denmark Odense Denmark

**Keywords:** ACE, ACE2, angiotensin II, GLP‐1, kidney, natriuresis, RAAS, sodium

## Abstract

Glucagon‐like peptide‐1 (GLP‐1) receptor agonists elicit cardiovascular and renal protection. In humans, GLP‐1 reduces plasma angiotensin II (ANGII) and increases renal perfusion, leading to increased natriuresis. The mechanisms underlying suppression of ANGII remain unclear but may involve ACE2 activation and/or a decrease in angiotensinogen. To address this hypothesis, we performed post‐hoc analyses on stored arterial plasma samples from a published study. The study used a randomized, placebo‐controlled cross‐over design in which eight healthy male adults ingested a sodium‐standardized diet for 4 days to reach steady state. Participants were examined during a 3‐h infusion of GLP‐1 (1.5 pmol/kg/min) or vehicle concurrent with an intravenous infusion of 0.9% NaCl (750 mL/h) to expand the extracellular volume. As previously published, GLP‐1 infusion significantly increased urinary sodium excretion, and plasma ANGII concentrations decreased significantly only during GLP‐1 infusion. The present analyses demonstrated that plasma angiotensinogen and ACE concentrations decreased similarly (parallel to renin concentrations) during GLP‐1 and vehicle. Plasma ACE2 and Ang‐(1–7) peptide concentrations remained unchanged during infusions of saline with and without GLP‐1. In conclusion, the acute ANGII‐lowering effect of GLP‐1 does not depend on changes in circulating concentrations of angiotensinogen, ACE, ACE2, or Ang‐(1–7). Changes in enzyme activities independent of concentrations cannot be excluded.

## 1 INTRODUCTION

Glucagon‐like peptide‐1 receptor agonist (GLP‐1RA) treatment elicits significant long‐term protection against negative cardiovascular and renal outcomes in patients with type 2 diabetes (Marso Bain, et al., 2016; Marso, Daniels, et al., 2016; Perkovic et al., 2024). In healthy humans, acute GLP‐1 infusion reduces plasma angiotensin II (ANGII) concentrations (Asmar et al., 2019, 2021; Skov et al., 2013) and primarily increases renal medullary perfusion, while also enhancing cortical perfusion and renal oxygenation during NaCl loading (Haddock et al., 2023).

GLP‐1 receptor expression in the human kidney is still not clarified. Thus, this interaction between GLP‐1 and ANGII – which is consistent across laboratories – appears increasingly important for explaining mechanisms for cardiovascular and especially renal long‐term protection (Hinrichs et al., 2024).

GLP‐1‐evoked natriuresis uncovered by expansion of the extracellular volume (ECV) in healthy individuals is associated with suppression of arterial plasma ANGII concentrations with no difference in arterial plasma renin, aldosterone, and ANP/BNP concentrations (Asmar et al., 2015, 2019, 2021). Suppression depends on GLP‐1 receptor activation but is not associated with changes in total renal plasma flow (RPF) and glomerular filtration rate (GFR) (Asmar et al., 2019, 2021).

This selective suppression of ANGII during GLP‐1 infusion with ECV expansion mimicking postprandial conditions is mechanistically not yet understood (Hinrichs et al., 2024). Fandino et al. (2018) demonstrated that liraglutide, a GLP‐1RA, stimulates angiotensin converting enzyme 2 (ACE2) expression within rat lung tissues whereas the potential effect on ACE is not known. An increase in counterregulatory ACE2 expression and/or a decrease in ACE expression would potentially lower ANGII concentrations. Furthermore, enhanced ACE2 activity contributes independently to cardiorenal protection (Jiang et al., 2014). Lower levels of circulating renin substrate (angiotensinogen) would also have a ANGII‐lowering effect.

**FIGURE 1:**
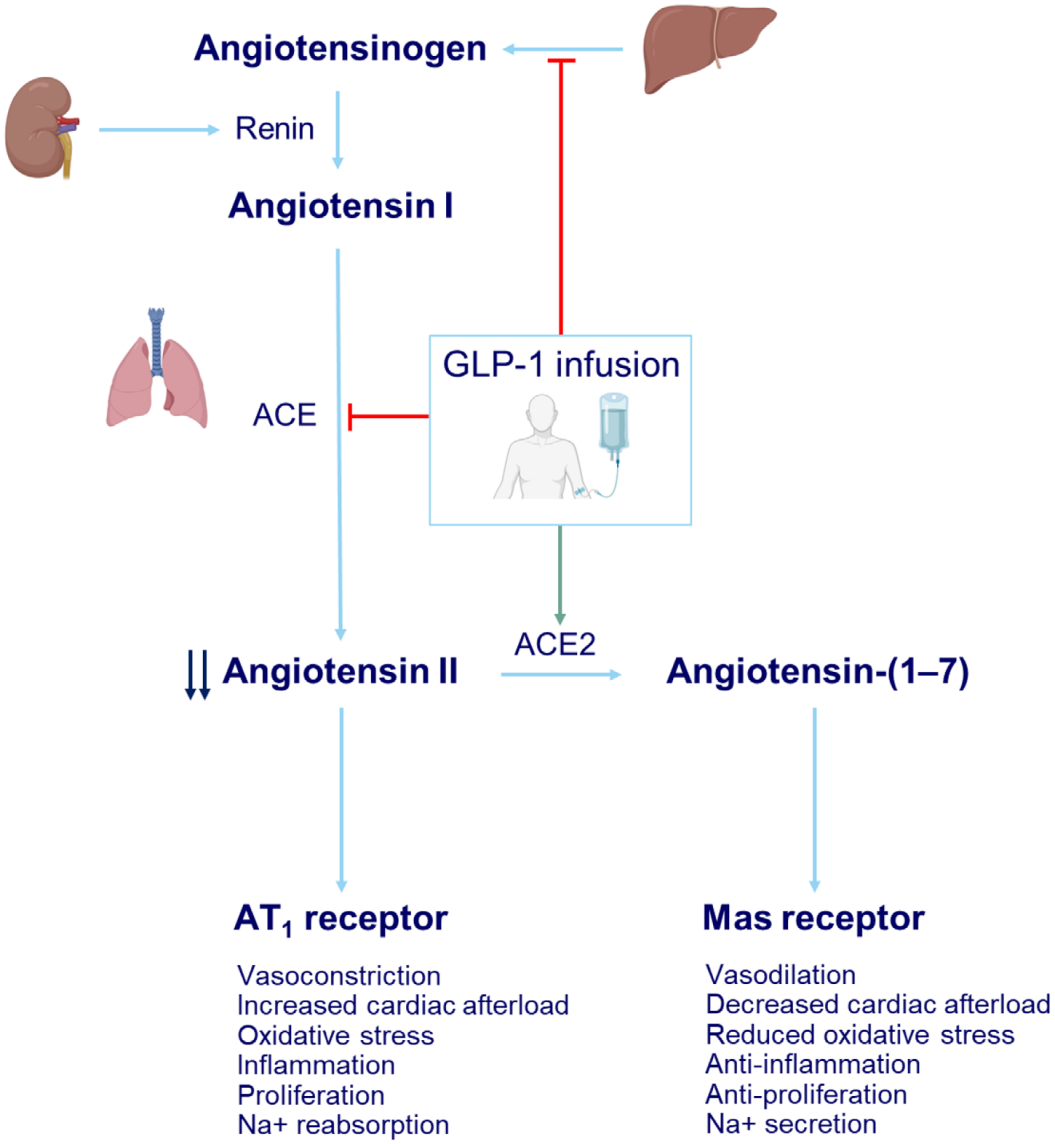
The renin‐angiotensin‐aldosterone system regulates cardiovascular and renal homeostasis through the interaction of two opposing pathways. The pressor pathway comprises the angiotensin converting enzyme (ACE), angiotensin II (ANGII), and the ANGII type 1 (AT_1_) receptor. Conversely, the depressor, or protective, pathway consists of angiotensin‐converting enzyme 2 (ACE2) and angiotensin‐(1–7) (Ang‐(1–7)) acting on the Mas receptor. Based on the selective decrease in circulating ANGII concentrations during GLP‐1 infusion, we hypothesize that GLP‐1 infusion in healthy humans lowers (red line) angiotensinogen and ACE and/or increases (green line) the counter‐regulatory ACE2/Ang‐(1–7), which in addition to reducing ANGII, contributes independently to cardiorenal protection (Jiang et al., 2014).

Our previous data (Asmar et al., 2019, 2021) and findings from preclinical models (Fandino et al., 2018) led us to hypothesize that GLP‐1 infusion in healthy humans increases ACE2 and Ang‐(1–7) plasma concentrations and/or lowers ACE and angiotensinogen plasma concentrations. These effects would account for the GLP‐1‐mediated selective decrease in circulating ANGII (Figure 1).

To address these hypotheses, we performed analyses *post‐hoc* on stored arterial plasma samples from a published study with acute GLP‐1 infusion reaching high‐physiological circulating levels (Asmar et al., 2019).

## 2 MATERIALS AND METHODS

The primary study objective was to investigate whether an ECV expansion would uncover a natriuretic effect of GLP‐1. Methods and results regarding this part have been described previously (Asmar et al., 2019). Consent to participate was obtained after the participants had read a description of the experimental protocol, which was approved by the Scientific Ethics Committee of the Capital Region of Copenhagen (H‐15004454) and conducted in accordance with the Declaration of Helsinki.

### 2.1 Design

In brief, a randomized, placebo‐controlled cross‐over design was applied in which participants ingested a standardized sodium diet (2822 kcal per day; 16% protein, 55% carbohydrate, 29% fat) for 4 days prior to each experiment, ensuring steady‐state.

### 2.2 Participants

Eight healthy male adults with mean age: 26 ± 3 years (SD) and lean body mass: 58.7 ± 5.5 kg were examined twice during a 3‐h infusion of either GLP‐1 (1.5 pmol/kg/min) or vehicle together with an intravenous infusion of 0.9% NaCl (750 mL/h) to expand the ECV.

### 2.3 Protocol

Participants fasted for 12 h before the beginning of the experiments. After emptying the bladder, confirmed by ultrasound, participants remained supine throughout the experiments and were given tap water (14 mL/kg, maximum 1000 mL) which was ingested within 10 min in order to keep participants in surplus of free water and thus suppress vasopressin at a constant low level.

During the experiments, urine collection was achieved by voluntary voiding together with timed bladder emptying immediately before the commencement of GLP‐1 or vehicle infusions and at the end of infusions. The total amount of urine collected during infusions was mixed and quantified separately. Bladder emptying was verified by ultrasound, and residual volumes were taken into account in subsequent calculations. Blood samples were drawn every 20 min during infusions.

Renal extraction of GLP‐1 was measured with catheters placed in the renal vein and radial artery. Renal plasma flow (RPF) and glomerular filtration rate (GFR) were measured via Fick's Principle using constant infusions of chromium‐51‐labeled EDTA (^51^Cr‐EDTA).

### 2.4 Materials for intravenous infusion

For the infusion, synthetic human GLP‐1 (7‐36amide, ≥97% purity; Bachem, Bubendorf, Switzerland, cat. no.: 4030663) was dissolved in saline with 0.5% human serum albumin (CSL Behring, Marburg, Germany) and identical to the natural human peptide by high‐performance liquid chromatography, mass, and sequence analysis. ^51^Cr‐EDTA was purchased from GE Healthcare (Brøndby, Denmark, cat. no.: CJ.13P).

### 2.5 Assays

Angiotensinogen (AGT) was measured by quantitative conversion to angiotensin I (ANG I) by prolonged incubation with added, excess, renin, followed by measurement of the formed ANG I by radioimmunoassay using the antibody‐trapping method (Poulsen & Jorgensen, 1974) as described in detail in (Kjolby et al., 2005). In brief, 50 μL human plasma was mixed with 100 μL anti‐ANGI antibody and 50 μL pure human renin (Rec. DNA, human type fra NIBSC 89/544). Samples incubated 24 h at 37°C. Next, the tubes incubated with ANG I tracer (^125^I‐ANG I; Glostrup Hospital, Glostrup, Denmark) at 4°C for 16–20 h, before secondary antibody (Sac‐Cel, Anti‐rabbit IgG, IDS, Boldon, UK) was added. Three hours later, the tubes were centrifuged, the supernatant was decanted and the sediment counted. The amount of ANG I (nmol/tube) was used to calculate the concentration of AGT in the plasma sample (μmol/L). The intra‐ and interassay coefficients of variation were 3 and 6%, respectively.

Arterial plasma ACE concentrations were measured using a commercial enzyme‐linked immunoassay kit (Human ACE ELISA Kit from Abcam, cat. no. Ab263889) as described by the manufacturer. Samples were diluted 1:10 and analyzed in duplicates. In house with a human plasma pool, the intra‐ and interassay coefficients of variation were 7.7% and 9.5%, respectively.

Arterial plasma ACE2 concentrations were measured using a commercial enzyme‐linked immunoassay kit (ACE2 human ELISA Kit, from AdipoGen Life Sciences, cat. no.: AG‐45B‐0023‐KI01) as described by the manufacturer. Samples were diluted 1:2 and analyzed in duplicates. The interassay coefficients of variation was 10.6%. Intraassay precision reported by the company is <5.2%.

Arterial plasma Ang‐(1–7) concentrations were measured, using a commercial enzyme immunoassay kit (Human Angiotensin 1–7 ELISA Kit, from Bio‐Techne, cat. no. NBP2‐69078) as described by the manufacturer. Samples were diluted 1:2 and analyzed in duplicates. The interassay coefficient of variation was 17%. Intraassay precision reported by the company is <5.4%.

### 2.6 Statistical analysis

Data were analyzed with GraphPad Prism 10 (GraphPad Software, Inc., La Jolla, CA). Area under the curve (AUC) was calculated via the trapezoidal rule, and the *t*‐test (two‐tailed) for paired data was used for comparing ΔAUC during GLP‐1 infusion and ΔAUC during vehicle. A correlation analysis between Δ ANGII and Δ urinary sodium excretion was conducted using Pearson's correlation coefficient. *p* < 0.05 was considered statistically significant.

## 3 RESULTS

As published (Asmar et al., 2019), GLP‐1 infusion increased urinary sodium and osmolar excretions significantly (two‐fold, *p* = 0.014). Arterial plasma renin concentrations decreased similarly during vehicle and GLP‐1 infusions. Arterial plasma ANGII concentrations decreased significantly only during GLP‐1 infusion by ~30%, *p* = 0.002 (Asmar et al., 2019). RPF and GFR remained unchanged on both days (Asmar et al., 2019).

There was no significant correlation between changes in urinary sodium excretions and arterial plasma ANGII concentrations during the 3‐h GLP‐1 infusion (r = 0.23, *n* = 8, *p* = 0.58).

**FIGURE 2:**
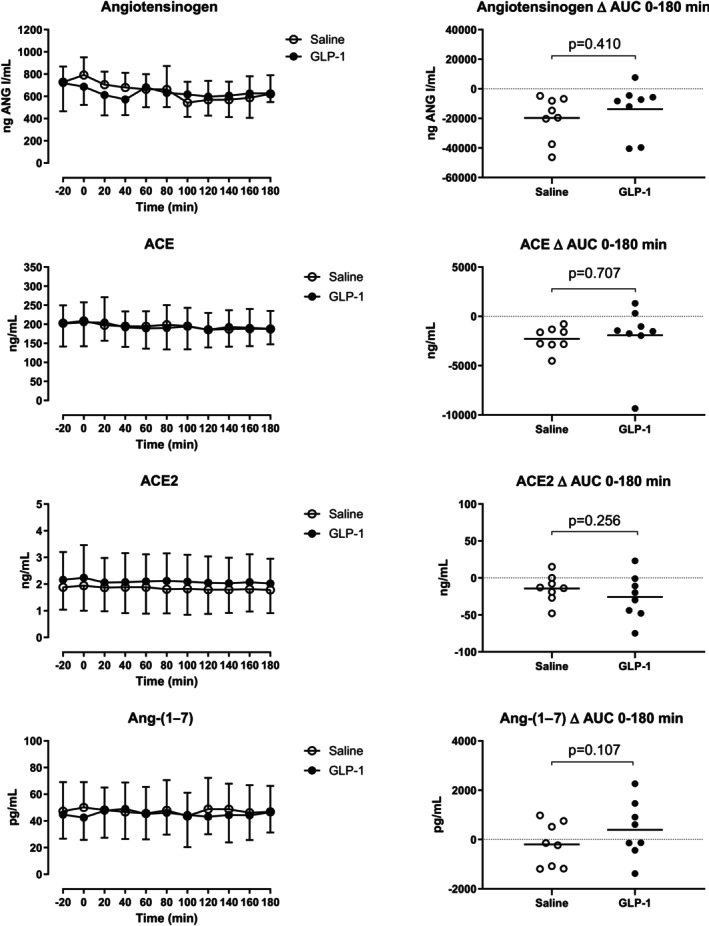
Arterial plasma concentrations of angiotensinogen, angiotensin‐converting enzyme (ACE), angiotensin‐converting enzyme 2 (ACE2), and angiotensin‐(1–7) (Ang‐(1–7). Left panel shows the time course of the plasma concentrations during the 180‐min infusion periods. Right panel shows the integrated effect during the infusions from 0 to 180 min compared to baseline levels. Data are presented as means ± SD.

Arterial plasma concentrations of angiotensinogen, ACE, ACE2, and Ang‐(1–7) are shown in Figure 2. Angiotensinogen and ACE enzyme concentrations decreased similarly and parallel to renin during GLP‐1 and vehicle infusions. ACE2 concentrations remained unchanged during infusions of vehicle and GLP‐1. Consistent with this, Ang‐(1–7) concentrations did not change on the two experimental days.

## 4 DISCUSSION

To our knowledge, this is the first human study to demonstrate that acute infusions of native GLP‐1 do not exert selective suppressive effects on circulating angiotensinogen concentrations available for cleavage by renin nor on circulating ACE concentrations. Also, the unchanged concentrations of ACE2 and Ang‐(1–7) indicate no increased degradation of ANGII along this pathway (Figure 1).

While our study found no changes in the circulating concentrations of angiotensinogen, renin, ACE or ACE2, the rapid suppression of ANGII suggests that alternative or localized regulatory pathways may be involved. Although the endocrine role of the renin‐angiotensin‐aldosterone system (RAAS) is indisputable, there is increasing evidence that its key component, ANGII, functions as a paracrine and intracrine peptide, likely via receptor‐mediated endocytosis (Ellis et al., 2012; Kobori et al., 2007; Paul et al., 2006).

One potential mechanism is the modulation of intrarenal RAAS activity. Preclinical studies have demonstrated that intrarenal ANGII blockade induces natriuresis independent of aldosterone and hemodynamics (Cervenka et al., 1998). This aligns with the GLP‐1‐induced selective ANGII suppression observed in the current study associated with a two‐fold increase in natriuresis (Asmar et al., 2019).

GLP‐1 receptors may be expressed in the renal vasculature and juxtaglomerular apparatus (Bjornholm et al., 2021; Jensen et al., 2015). Activation of these receptors may lead to a direct, localized inhibition of ANGII production within the kidney that is not fully reflected by systemic enzyme concentrations (Jimenez et al., 2019).

Furthermore, the inhibition of the sodium‐hydrogen exchanger 3 (NHE3) in the proximal tubule may be involved in the GLP‐1‐induced natriuresis which increases sodium delivery to the macula densa (Gutzwiller et al., 2004; Skov et al., 2013). This may activate tubuloglomerular feedback (TGF), which can acutely suppress the local generation of ANGII. It is also possible that GLP‐1 enhances the activity of alternative ANGII‐degrading proteases, such as neprilysin, which were not measured in this study (Mentlein, 2009). Lastly, GLP‐1 has been shown to activate the cAMP‐PKA pathway, which can directly interfere with ANGII signaling and synthesis at the cellular level (Lee et al., 2024; Marzook et al., 2021).

From a pathophysiological perspective, it is interesting that circulating and intrarenal ANGII generally promote the progression of diabetic nephropathy and hypertensive kidney disease through proinflammatory and growth‐promoting effects (Ellis et al., 2012; Kobori et al., 2007). Additionally, the GLP‐1‐associated suppression of ANGII concomitant with increased natriuresis is dependent on GLP‐1R activation (Asmar et al., 2021). This ANGII suppression possibly accounts for the GLP‐1‐mediated increase in mainly renal medullary perfusion but also renal cortical perfusion and renal oxygenation in the healthy human kidney during saline loading (Haddock et al., 2023).

Renal hypoxia plays a central role in the pathway for most etiologies of chronic kidney disease, including microvascular damage and excessive renal energy expenditure driven by hyperglycemia‐related glomerular hyperfiltration and associated sodium reabsorption (Andersen et al., 2023; Fine & Norman, 2008). Thus, by preserving renal tissue oxygenation, improved renal perfusion may contribute to the long‐term cardiorenal benefits of GLP‐1RAs. Despite the acute design of the present study, the GLP‐1 infusion rate utilized in this study (1.5 pmol/kg/min) was chosen to achieve steady‐state plasma concentrations in the high‐physiological to low‐pharmacological range (Asmar et al., 2015, 2020). These levels are comparable to the peak plasma concentrations of the estimated fraction of unbound (free) GLP‐1RAs (Jensen et al., 2018).

### 4.1 Limitations

First, the enzyme activities for ACE and ACE2 were not determined; this remains a relevant parameter, as activity could change even with constant enzyme concentration. Factors of known significance for enzyme activity are chloride concentration, pH (which did not change), zinc, oxidative stressors, glycosylation, and nitrosylation.

Furthermore, plasma levels of ACE and ACE2 are surrogate measures likely reflecting integrated and variable cellular loss and shedding from high‐abundance tissues such as the endothelium and, for ACE2, the renal and intestinal luminal epithelium. Consequently, tissue levels in certain areas could change without corresponding changes in plasma levels.

The ECV expansion on both days could generally contribute to a decrease in plasma concentrations of small peptides distributed in ECV. However, this expansion cannot explain differential effects on the decreasing ANGII levels and the constant Ang‐(1–7) levels.

Finally, a major limitation of this study is that it was originally designed with urinary sodium excretion as the primary endpoint and analyses of angiotensinogen, ACE, ACE2, and Ang‐(1–7) were conducted post hoc. Nevertheless, the GLP‐1‐associated selective ANGII suppression in the present study was highly significant (~30%, *p* = 0.002) compared to vehicle (Asmar et al., 2019).

In conclusion, it remains a challenge to understand how GLP‐1, acting through its cognate receptor, lowers selectively circulating ANGII (Asmar et al., 2019, 2021) in the face of unchanged concentrations of other enzymes in the RAAS.

## AUTHOR CONTRIBUTIONS

A.A. and B.L.J. conceived and designed research. A.A. performed experiments. A.A., B.L.J., M.A., C.M.S., and J.K.A. analyzed data. A.A., B.L.J., M.A., C.M.S., and J.K.A. interpreted results of experiments. A.A. prepared figures. A.A. and B.L.J. drafted manuscript. A.A., B.L.J., M.A., C.M.S., and J.K.A edited and revised manuscript. A.A., B.L.J., M.A., C.M.S., and J.K.A approved final version.

## FUNDING INFORMATION

This study was supported financially by The Danish Heart Foundation, The Board of Research of Bispebjerg University Hospital, The Arvid Nilssons Foundation, and The Dagmar Marshalls Foundation.

## CONFLICT OF INTEREST STATEMENT

The authors declare no conflict of interest.

## ETHICS STATEMENT

Consent to participate was obtained after the participants had read a description of the experimental protocol, which was approved by the Scientific Ethics Committee of the Capital Region of Copenhagen (H‐15004454) and conducted in accordance with the Declaration of Helsinki.

## Data Availability

All data generated or analyzed during this study are included in this published article.
